# The collaborative cross strains and their founders vary widely in cocaine-induced behavioral sensitization

**DOI:** 10.3389/fnbeh.2022.886524

**Published:** 2022-10-05

**Authors:** Sarah A. Schoenrock, Leona Gagnon, Ashley Olson, Michael Leonardo, Vivek M. Philip, Hao He, Laura G. Reinholdt, Stacey J. Sukoff Rizzo, James D. Jentsch, Elissa J. Chesler, Lisa M. Tarantino

**Affiliations:** ^1^Department of Genetics, School of Medicine, Chapel Hill, NC, United States; ^2^Center for Systems Neurogenetics of Addiction, Bar Harbor, ME, United States; ^3^The Jackson Laboratory, Bar Harbor, ME, United States; ^4^Department of Medicine, University of Pittsburgh, Pittsburgh, PA, United States; ^5^Department of Psychology, Binghamton University, Binghamton, NY, United States; ^6^Division of Pharmacotherapy and Experimental Therapeutics, Eshelman School of Pharmacy, University of North Carolina at Chapel Hill, Chapel Hill, NC, United States

**Keywords:** mice, cocaine, collaborative cross, inbred, sensitization, genetics, heritability

## Abstract

Cocaine use and overdose deaths attributed to cocaine have increased significantly in the United States in the last 10 years. Despite the prevalence of cocaine use disorder (CUD) and the personal and societal problems it presents, there are currently no approved pharmaceutical treatments. The absence of treatment options is due, in part, to our lack of knowledge about the etiology of CUDs. There is ample evidence that genetics plays a role in increasing CUD risk but thus far, very few risk genes have been identified in human studies. Genetic studies in mice have been extremely useful for identifying genetic loci and genes, but have been limited to very few genetic backgrounds, leaving substantial phenotypic, and genetic diversity unexplored. Herein we report the measurement of cocaine-induced behavioral sensitization using a 19-day protocol that captures baseline locomotor activity, initial locomotor response to an acute exposure to cocaine and locomotor sensitization across 5 exposures to the drug. These behaviors were measured in 51 genetically diverse Collaborative Cross (CC) strains along with their inbred founder strains. The CC was generated by crossing eight genetically diverse inbred strains such that each inbred CC strain has genetic contributions from each of the founder strains. Inbred CC mice are infinitely reproducible and provide a stable, yet diverse genetic platform on which to study the genetic architecture and genetic correlations among phenotypes. We have identified significant differences in cocaine locomotor sensitivity and behavioral sensitization across the panel of CC strains and their founders. We have established relationships among cocaine sensitization behaviors and identified extreme responding strains that can be used in future studies aimed at understanding the genetic, biological, and pharmacological mechanisms that drive addiction-related behaviors. Finally, we have determined that these behaviors exhibit relatively robust heritability making them amenable to future genetic mapping studies to identify addiction risk genes and genetic pathways that can be studied as potential targets for the development of novel therapeutics.

## Introduction

Cocaine remains one of the most used illicit substances worldwide and use is particularly high in the United States (Peacock et al., 2018; Vachiery et al., 2019). Cocaine use in the US has increased significantly over the last 10 years as have overdose deaths attributed to the drug (John and Wu, 2017; Kariisa et al., 2019). The personal and societal burdens imposed by cocaine use disorders (CUDs) are staggering and relatively few effective treatment options exist. Not all who use cocaine will develop a CUD, suggesting that there are individual differences in risk. Identifying and understanding the mechanisms that contribute to risk will drive development of novel treatments and prevention strategies.

Twin studies and genome-wide association studies in humans support a significant role for genetic factors in CUD risk (Goldman et al., 2005; Ducci and Goldman, 2012; Gelernter et al., 2014). To date, however, very few causal genes have been identified in human studies. Animal models have provided important insights into the genetic and neurobiological pathways associated with drug response and reward (Russo et al., 2010; Robison and Nestler, 2011; Charbogne et al., 2014; Simmler and Blakely, 2019; Solinas et al., 2019). These experimental systems are a valuable resource that enable examination of drug effects from the first exposure in naïve animals through more extended exposure paradigms that model the rewarding, reinforcing and motivational properties of drugs. Intravenous drug self-administration (IVSA) protocols are generally considered the “gold standard” for modeling addiction in rodent models. However, IVSA is technically challenging in mice, is labor- and time-intensive and often difficult to establish. Since almost all drugs of abuse increase locomotor activity and there is considerable overlap in dopaminergic mechanisms involved in both psychostimulant-induced locomotor activation and drug reward pathways (Cornish and Kalivas, 2001), drug-induced locomotor activation protocols have been used extensively in rodent addiction research.

Locomotor activation following acute drug exposure is frequently used as a rodent-based assay to characterize the biological basis of initial drug sensitivity. In humans, initial sensitivity predicts future drug use but a positive association between initial sensitivity and the development of a substance use disorder has not been well-established (de Wit and Phillips, 2012). Augmented locomotor activation observed upon repeated exposures, termed behavioral sensitization, is frequently used in model organisms as an indirect measure of underlying neural adaptations that occur in response to chronic drug use (Vanderschuren and Kalivas, 2000; Cornish and Kalivas, 2001; Thomas et al., 2001, 2008; Steketee, 2005; Steketee and Kalivas, 2011). Previous work has established two distinct components or phases of behavioral sensitization at the pharmacological and neuroanatomical levels: initiation/development and expression/maintenance of the sensitized behavioral response (Kalivas and Stewart, 1991; Pierce and Kalivas, 1997; Vanderschuren and Kalivas, 2000). The neural adaptations that occur upon repeated exposure to the drug across these phases and the behavioral changes that result have been shown to persist long after withdrawal and are thought to underly continued and persistent drug-taking and -seeking behavior in addicted individuals leading to a high risk of relapse (Paulson et al., 1991; Cornish and Kalivas, 2001; Steketee and Kalivas, 2011).

Although not universally observed (Yamamoto et al., 2013), numerous studies have reported a positive relationship between IVSA and behavioral sensitization to psychostimulants. Repeated exposure to sensitizing doses of psychostimulants enhances acquisition of drug self-administration (Piazza et al., 1990; Yamamoto et al., 2013) and sensitizes rats to the rewarding effects of cocaine (Horger et al., 1990; Schenk and Partridge, 2000). Conversely, repeated psychostimulant exposure during self-administration results in sensitized locomotor response to the drug (Hooks et al., 1994; Phillips and Di Ciano, 1996; De Vries et al., 1998; Zapata et al., 2003). Moreover, rats that are pre-exposed to amphetamine, either systemically or directly into the ventral tegmental area, work harder to obtain the drug under a progressive ratio schedule of reinforcement (Vezina et al., 2002). Although the relationship between behavioral sensitization and IVSA is likely complex and will vary across different populations and genetic backgrounds, these studies support the use of locomotor response to acute and repeated drug exposures as an accessible paradigm for studying genetic and neurobiological mechanisms that influence drug reward and response. In fact, studying behavioral sensitization in diverse genetic backgrounds can reveal relationships among addiction-relevant behaviors that have, as yet, gone undiscovered.

Genetic loci that influence cocaine-induced acute locomotor sensitivity and behavioral sensitization have been identified in numerous studies. However, these behaviors have been examined in very few genetic backgrounds, limiting the general applicability and translatability of the findings (Tolliver et al., 1994; Miner and Marley, 1995; Phillips et al., 1998; Jones et al., 1999; Boyle and Gill, 2001, 2009; Gill and Boyle, 2003, 2008; Kumar et al., 2013; Saul et al., 2020). The Collaborative Cross (CC) is a panel of recombinant inbred lines derived from intercrossing 5 classical and 3 wild-derived inbred mouse strains that, collectively, capture almost 90% of the known genetic diversity in laboratory mice (Threadgill and Churchill, 2012). Surveying the phenotypic diversity present in the CC offers the opportunity to explore complex behaviors on a genetically defined and reproducible population of inbred mouse strains that have been optimized for systems genetics studies.

We assessed cocaine-induced locomotor sensitivity and behavioral sensitization in a set of 51 CC strains and their 8 inbred founders. These genetic reference populations have distinct advantages for the investigation of complex traits. Together these inbred populations are ideal for investigating trait correlations, establishing the genetic architecture of quantitative traits, and estimating trait heritabilities. Moreover, phenotypic investigation of a panel of CC mice can yield strains with extreme or abnormal phenotypes that can serve as disease models for mechanistic and pharmacologic studies (Saul et al., 2019). In this study, we also derived discrete phenotypic variables that will be used for future genetic mapping and gene expression studies in the Diversity Outbred (DO) mouse population. The DO population is derived from the same 8 CC founder strains and is maintained as an outbred population that is ideally suited to gene co-expression analyses and genetic mapping studies (Gatti et al., 2014).

Our data highlight the significant phenotypic variation present for acute and sensitized cocaine-induced locomotor response in this genetically heterogeneous population. The availability of these data in the CC and founder strains affords the opportunity to assess the relationship of these behaviors with other phenotypes as more data are collected and made public in this valuable genetic resource.

## Materials and methods

### Mice

Cohorts of male and female A/J, C57BL/6J, 129S1/SvlmJ, NOD/ShiLtJ, NZO/HlLtJ, CAST/EiJ, PWK/PhJ, WSB/EiJ, and CC strains were obtained from The Jackson Laboratory (JAX) Genetic Resource Sciences for behavioral testing in the Center for Systems Neurogenetics of Addiction (CSNA) Behavioral Phenotyping Core. Mice at JAX were housed in specific pathogen-free facilities on a 12-hr light/dark cycle with lights on at 6:00 A.M. Food (NIH31 5K52 chow, LabDiet/PMI Nutrition, St. Louis, MO) and water were provided *ad libitum* throughout the experiment. Animals were singly housed starting at 6 weeks of age until the completion of behavioral testing. Prior to behavioral sensitization, mice were tested in a 4-day battery that included open field, light/dark, holeboard, and novelty place preference assays [data and methods described in Saul et al. (2020) and will not be discussed here]. Detailed standard operating procedures for these behavioral assays can be found at https://phenome.jax.org/projects/CSNA03/protocol. Mice were moved to an anteroom for acclimation for a minimum of 30 min prior to behavioral testing by a team of animal handlers blind to strain; testing by each handler was randomized across batches and strains. *All mice at JAX were tested between the hours of 7 A.M. and 3 P.M.* Mice tested at JAX averaged 78 days of age at the time of testing (range 49–143 days).

Separate cohorts of male and female A/J, C57BL/6J, 129S1/SvlmJ, NOD/ShiLtJ, NZO/HlLtJ, CAST/EiJ, PWK/PhJ, WSB/EiJ mouse strains were purchased from the JAX and transported to the University of North Carolina (UNC) for behavioral sensitization testing. Male and female CC016/GeniUncJ, CC061/GeniUncJ, and CC074/UncJ mice were purchased from the UNC Systems Genetics Core Facility. All mice were group housed in a specific pathogen-free facility at UNC and maintained on a 12-hr light/dark cycle with lights on at 7:00 A.M. Food (Harlan Teklad 2920, Envigo, Frederick, MD, United States) and water were provided *ad libitum* throughout the experiment. Mice tested at UNC were behaviorally naïve prior to behavioral sensitization tests and were transported directly from the animal holding room to the procedure space immediately prior to testing. All mice at UNC were tested between the hours of 8 A.M. and 12 P.M. by the same animal handler. Mice tested at UNC averaged 75 days of age at the time of testing (range 70–100 days). Numbers of mice per strain, treatment group and sex tested at both sites are provided in Supplementary Table 1.

All procedures conducted at JAX and UNC were approved by the Institutional Animal Care and Use Committees at each respective institution and followed guidelines set forth by the National Institutes of Health Guide for the Care and Use of Laboratory Animals, 8th Edition.

### Drugs

Cocaine was prepared fresh each test day at a volume 1 mg/mL by dissolving cocaine hydrochloride (Sigma-Aldrich, St. Louis, MO) in 0.9% saline. Cocaine was administered by intraperitoneal (i.p.) injection at a dose of 10 mg/kg in a volume of 0.1 mL/10 g of body weight. Saline injections were also administered by i.p. injection at the same volume. Body weight was determined for each mouse on each day the drug was administered to ensure accurate dosing.

### Open field apparatus

The open field (OF) arenas were 43.2 × 43.2 × 33 cm with white Plexiglas floors and clear Plexiglas walls (ENV-515-16, Med Associates, St. Albans, VT, United States). Infrared photobeam sensors at 2.54 cm intervals on the x, y, and z-axes tracked the animals’ position and activity automatically during testing. Each OF was enclosed in a sound-attenuating cabinet (73.5 × 59 × 59 cm) illuminated by two incandescent lights, each affixed in the upper rear two corners of the cabinet at a height of approximately 18.5 inches from the center of the arena floor which provided an illumination of 60 ± 10 lux when measured in the center of arena floor.

### Behavioral sensitization to cocaine

Behavioral sensitization to cocaine was tested in the open field arena (described above). See Table 1 for a description of test days and treatment groups. Our behavioral sensitization procedure was based on our previously published methods (Schoenrock et al., 2020) and modified from previously published literature (Phillips et al., 2010; Kumar et al., 2013). Mice were tested for 90 min each test day in a 19-day protocol. Mice were placed into the open field for 30 min, removed and injected intraperitoneally with either saline or cocaine and returned to the arena for 60 min. The CC and founder strain surveys included two test groups—cocaine-treated and saline-treated. Mice in the saline treatment group received only saline injections on each test day. Mice in the cocaine-treatment group received saline injections on test days 1, 2, and 12 and cocaine injections (10 mg/kg) on test days 3, 5, 7, 9, 11, and 19. This dose of cocaine has been shown in our previous studies and by others to reliably produce behavioral sensitization across different inbred strains of mice (Phillips et al., 2010; Kumar et al., 2013; Schoenrock et al., 2020). All other days were non-testing days and mice were not handled on those days. Total distance moved in the 60-min post injection was used for all data analyses and to derive behavioral measures outlined in Table 2. *Rearing behavior (number of rearings) was also measured in the open field and is publicly available in the Mouse Phenome Database.^1^*

**TABLE 1:** Behavioral sensitization protocol.

DAY	1	2	3	4	5	6	7	8	9	10	11	12	19
**Drug-exposed group**	SAL	SAL	**COC**	No testing	**COC**	No testing	**COC**	No testing	**COC**	No testing	**COC**	SAL	**COC**
**Saline-exposed group**	SAL	SAL	SAL		SAL		SAL		SAL		SAL	SAL	SAL

SAL, Saline; COC, Cocaine.

**TABLE 2:** Derived variables calculated from 19-day behavioral sensitization data.

Behavioral variable	Description (derived variable)
Day 1 locomotion	Distance traveled on 1st exposure to open field and saline injection (Day 1)
Day 2 locomotion	Distance traveled on 2nd exposure to open field and saline injection (Day 2)
Habituation	Change in locomotor activity from Day 1 to 2 (Day 2–1)
Initial locomotor response	Locomotor response after 1st exposure to cocaine (Day 3–2)
Initial locomotor sensitization	Distance traveled after 2nd cocaine exposure (Day 5–3)
Locomotor sensitization	Area under the curve (AUC) of distance across 5 consecutive cocaine exposures (Day 3,5,7,9,11)
Sensitization expression	Locomotor response to cocaine after 1-week withdrawal (Day 19–11)
Conditioned activation	Locomotor response to saline after repeated cocaine exposures (Day 12–2)

### Statistical analyses

Open field behavioral data were analyzed post-session using commercially available software (Activity Monitor 7.06, Med Associates). All statistical analyses were performed using R Studio 1.2.13335 or SPSS Statistics v26 for Mac OS X 10.6 +. Graphs were generated using Graphpad Prism 8 for Mac OS X. Descriptive statistics were examined across all 19 days of testing for each strain and each study (cocaine vs. saline exposure). *For founder and CC strain analyses, a three-way linear mixed effects ANOVA was performed on residuals saved after regressing out variance due to sex. The ANOVA was performed using the R package lmerTest to evaluate the fixed effects of treatment, strain and day on day-specific trait measures with a random effect of mouse.* Significant differences involving treatment or strain were further evaluated by *post hoc* Tukey’s HSD. Significance for all comparisons was set at α = 0.05. ANOVA was also performed within strains to assess replicability of locomotor activity across testing sites with site, day and sex as independent variables. Significant main effects were examined by *post hoc* Tukey’s HSD. *Inbred strain means were used to perform Pearson bivariate correlation analyses to assess relationships among all behavioral variables. Behavioral variables were also correlated with body weight as determined on the first day of behavioral testing. Within- and between-strain phenotypic variances were used to estimate the heritability and determine the proportion of phenotypic variation that is due to genetic vs. non-genetic factors.* Broad sense heritability (H^2^) values were calculated in the cocaine-exposed group only using the following equation: [Mean Square Strain Effects/(Mean Square Strain Effect + (Mean Number of Mice per Strain-1)*Mean Square Residuals)]. *We implemented model-based bootstrapping to obtain the standard error for an estimate of the heritability. New observations were simulated based on the same model parameters of the original fitted model. Then the model was re-fitted using the new observations and the heritability was re-calculated. The process was repeated 1,000 times to calculate the standard error for the estimate of the heritability.*

## Results

### Treatment and strain effects

**FIGURE 1:**
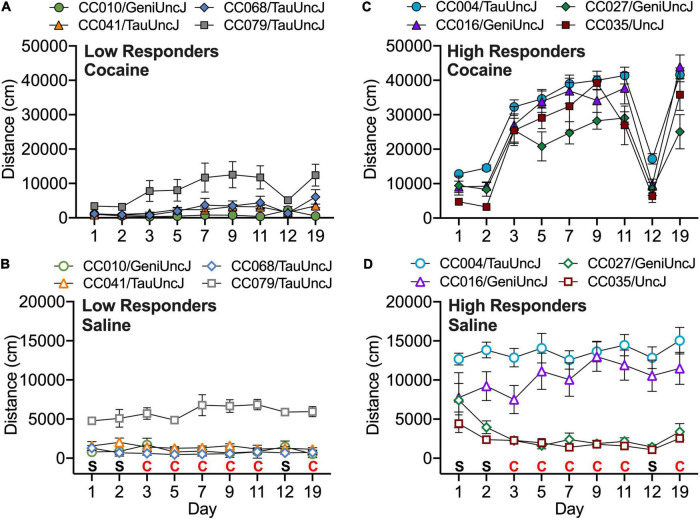
Strain means across the 19-day protocol are shown for Collaborative Cross strains with the lowest **(A)** and highest **(C)** responses to 10 mg/kg cocaine (filled symbols). Response to saline exposure in the same strains is shown in **(B,D)** (open symbols). Error bars are standard error of the mean.

A linear mixed model was used to test the effects of test day, strain and treatment (cocaine vs. saline) on locomotor activity in CC and founder strains across all 19 days of testing. *In order to account for sex differences, the variance due to sex was regressed out and residuals were used as an input in the linear mixed model.* Significant day, treatment and strain as well as day × treatment, day × strain, treatment × strain and day × treatment × strain interaction effects were observed (all *p* < 2 × 10^–16^). *Post hoc analyses of treatment effects revealed that 35 of the 58 strains showed a significant increase in locomotor activity when exposed to cocaine vs. saline* (Supplementary Table 2). *CC strains that were most significantly activated in response to cocaine include CC004/TauUncJ, the strain identified in our previously published study* ((Schoenrock et al., 2020)*; p* = *5.9* × *10^–28^), as well as CC016/GeniUncJ (p* = *1.1* × *10^–9^), CC027/GeniUncJ (p* = *3.6* × *10^–7^) and CC035/UncJ (p* = *5.4* × *10^–9^). Several CC strains were also identified as non-responders including CC041/TauUncJ (p* = *0.62) also described in our previously published study* (Schoenrock et al., 2020), *as well as CC010/GeniUncJ (p* = *0.95), CC068/TauUncJ (p* = *0.66) and CC079/TauUncJ (p* = *0.62)* (Figure 1).

We also assessed treatment and strain effects on derived variables (Table 2 and Supplementary Table 3). We observed significant strain effects for Day 1 and Day 2 locomotor activity (both *p* < 2 × 10*^–^*^16^), habituation (Day 2–1; *p* = 4.3 × 10*^–^*^6^), initial locomotor response to the first dose of cocaine (Day 3–2; *p* < 2 × 10*^–^*^16^), initial sensitization (Day 5–3; *p* = 0.002) and sensitization area under the curve (AUC; *p* = 1.1 × 10*^–^*^4^). No significant strain effects were observed for sensitization expression (Day 19–11; *p* = 0.061) or conditioned activation (Day 12–2; *p* = 0.38). Significant effects of treatment were observed for all derived variables (all *p* < 0.05) with the exception of habituation (Day 2–1; *p* = 0.34; Supplementary Table 3). There was a significant strain by treatment interaction for acute locomotor response to cocaine (Day 3–2; *p* < 2 × 10*^–^*^16^), initial sensitization (Day 5–3; *p* = 0.025) and sensitization AUC (*p* = 0.013).

### Sex effects in the collaborative cross founder strains

**FIGURE 2:**
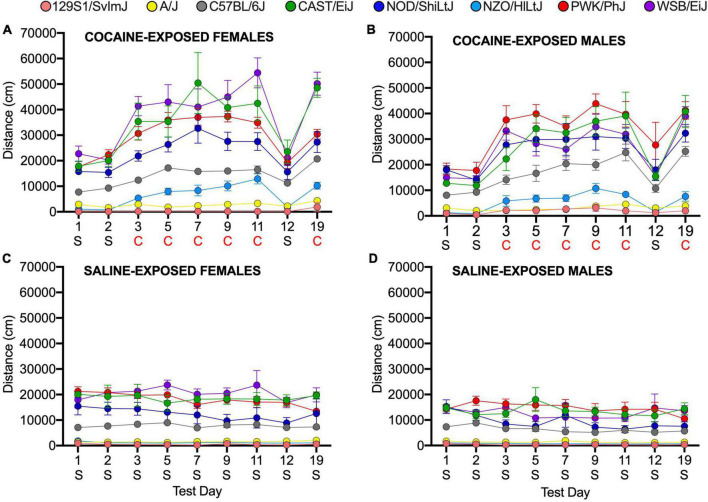
Locomotor activity in response to cocaine and saline in female **(A,C)** and male **(B,D)** founder strain mice. S, Saline; C, Cocaine. Error bars are standard error of the mean.

We assessed sex differences and strain by sex interactions in the founder strains using a linear mixed model. We observed a significant main effect of sex in the founder group; female mice are, overall, more active than male mice (*p* = 0.004). We also observed significant sex by strain effects (*p* = 1.4 × 10^–4^). CAST/EiJ females are significantly more active than CAST/EiJ males (*p* = 4.8 × 10^–3^) as are WSB/EiJ females in comparison to WSB/EiJ males (*p* = 2.8 × 10^–6^). No significant sex differences were observed in the remaining six founder strains (Figure 2, Supplementary Table 4 and Supplementary Figure 2). We did not identify any significant sex by treatment or sex by day effects.

### Heritability

**FIGURE 3:**
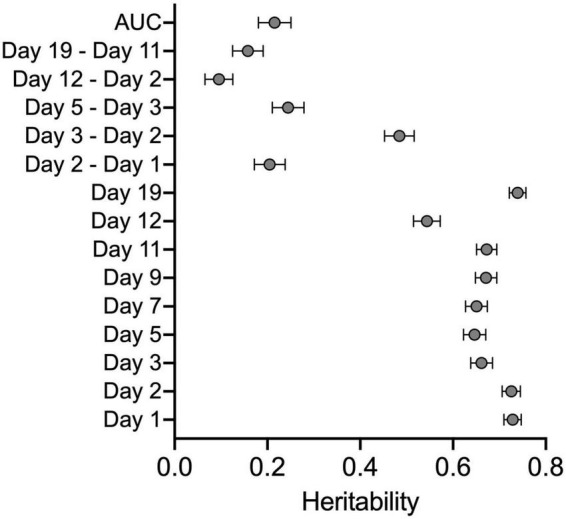
Broad sense heritability estimates for all derived variables and locomotor activity across all days of testing. Error bars are SEM.

Derived variables were used to generate CC and founder strain means. These data were used to calculate broad sense heritability in the cocaine-treated group to determine the proportion of phenotypic variation due to genetic background. Heritabilities ranged from 0.10 to 0.73 (Figure 3 and Supplementary Table 5A). The highest heritabilities were observed for locomotor activity on Days 1 and 2 (both H^2^ = 0.73) and acute locomotor response to cocaine (Day 3–2; H^2^ = 0.48). Initial sensitization (Day 5–3), behavioral sensitization (AUC) and habituation (Day 2–1) all had similar heritabilities of 0.25, 0.22, and 0.22, respectively. Sensitization expression (Day 19–11) and conditioned activation (Day 12–2) had the lowest heritabilities (H^2^ = 0.16 and 0.10, respectively).

### Correlation of sensitization behaviors

Correlation of locomotor activity on all testing days as well as derived variables (Table 2) was performed using CC and founder strain means in cocaine-exposed groups only. Correlation coefficients are shown in (Table 3 and Supplemental Figure 3) and both coefficients and unadjusted *p*-values are shown in Supplementary Table 6. We avoided autocorrelations between derived variables and individual days or variables that included the days encompassed by the derived measure. We detected significant positive correlations between locomotor activity (either saline- or cocaine-induced) and all single days of testing (all *p* < 0.001). Acute locomotor response to cocaine (Day 3–Day 2) was also significantly and positively correlated with activity following saline exposure on Days 1 and 12 as well as activity following cocaine exposure on Days 5, 7, 9, 11, and 19. Behavioral sensitization (AUC) was significantly and positively correlated with conditioned activation (D12-D2; *p* = 3.0 × 10^–4^) and expression of sensitization on Day 19 (*p* = 2.0 × 10^–5^). Conditioned activation was also significantly correlated with initial sensitization (D5–D3; *p* = 3.1 × 10^–5^). We observed no significant correlation of habituation (D2–D1) with any other sensitization variable. Body weight did not correlate not any behavioral variables (Table 3).

**TABLE 3:** Pearson correlation coefficients among all measures in CC and founder strains.

Day 2	0.95														
Day 2–1															
Day 3	0.81	0.83	0.17												
Day 3 - Day 2	0.54														
Day 5	0.74	0.76	0.17	0.95	0.89										
Day 5–3	0.16	0.16	0.04												
Day 7	0.71	0.75	0.22	0.93	0.86	0.95	0.50								
Day 9	0.71	0.73	0.17	0.93	0.88	0.96	0.51	0.97							
Day 11	0.73	0.75	0.17	0.93	0.86	0.94	0.46	0.95	0.97						
AUC	0.14	0.16	0.09												
Day 12–2	0.08			0.23		0.37	0.51	0.33	0.37	0.35	0.45				
Day 12	0.87	0.91	0.24	0.83	0.59	0.82	0.35	0.79	0.79	0.81	0.32				
Day 19–11	0.10	0.12	0.11	0.29	0.34	0.29	0.14	0.28	0.24			−0.03	0.10		
Day 19	0.70	0.72	0.19	0.92	0.88	0.94	0.46	0.94	0.95	0.97	0.52	0.31	0.76		
Weight	−0.33	−0.33	−0.09	−0.38	−0.33	−0.32	0.01	−0.29	−0.27	−0.29	0.06	0.02	−0.30	−0.05	−0.28
	Day 1	Day 2	Day 2–1	Day 3	Day 3–2	Day 5	Day 5–3	Day 7	Day 9	Day 11	AUC	Day 12–2	Day 12	Day 19–11	Day 19

Correlations among individual days and derived variables that contain the same day as part of their calculation are not reported. Correlations attaining significance at Bonferroni corrected *p* < 0.05 are noted by green shading and bold type.

### Replicability of sensitization behavior

#### Founder replicability

**FIGURE 4:**
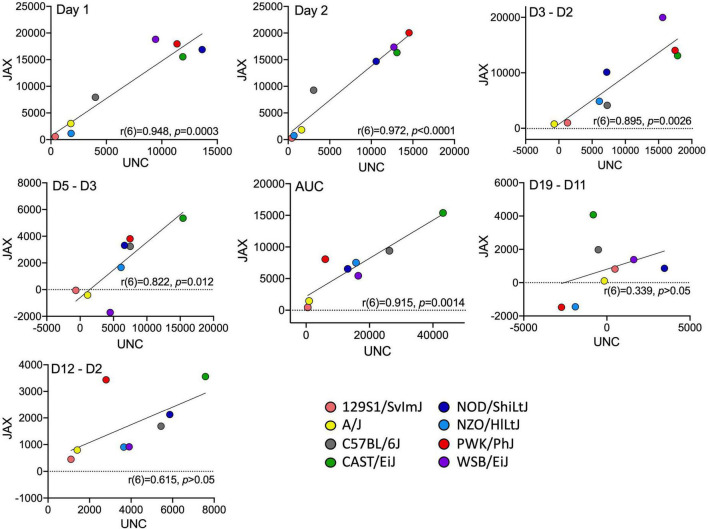
Correlation of founder strain means for all derived variables for mice tested at UNC and JAX. Each data point represents a strain mean. The line of best fit and correlation coefficient (r) with *p*-value are shown for each derived variable.

**FIGURE 5:**
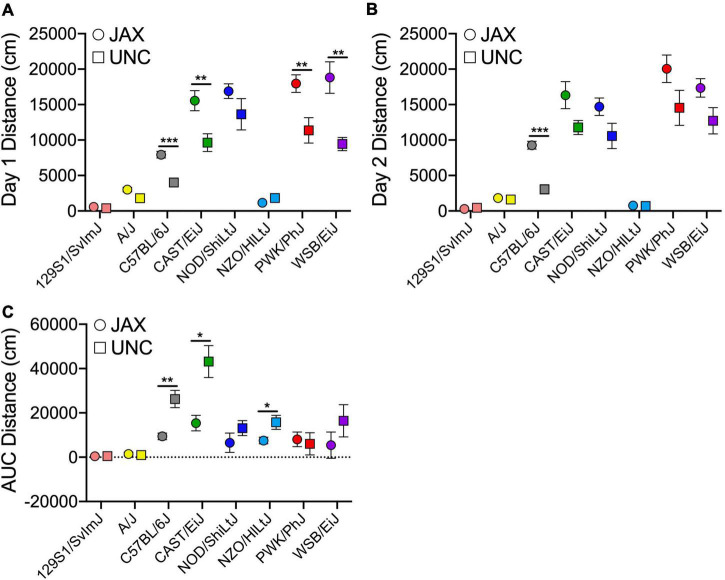
Founder strain means for distance on Day 1 (A) and Day 2 (B) and sensitization AUC (C) measured at JAX (circles) and UNC (squares). Error bars are standard error of the mean. *p < 0.05, **p < 0.01, ***p < 0.001.

A separate cohort of cocaine-exposed mice from the founder strains were tested at UNC (Supplementary Table 1). Comparison of the derived variables measured in the UNC cohort with the cocaine-exposure group of mice tested at JAX allows us to assess the replicability of sensitization behavior across the two sites (Figure 4). ANOVA of each behavioral measure with strain, sex and test site as independent factors yielded significant strain effects for Day 1 [*F*_(7_, _153)_ = 69.1; *p* = 3.4 × 10^–44^] and habituated locomotor activity [Day 2; *F*_(7_, _151)_ = 68.9; *p* = 7.7 × 10^–44^], acute locomotor response to cocaine [Day 3–Day 2; *F*_(7_, _151)_ = 19.0; *p* = 4.7 × 10^–18^], initial sensitization [Day 5–Day 3; *F*_(7_, _150)_ = 5.8; *p* = 6.0 × 10^–6^] and behavioral sensitization [AUC; *F*_(7_, _146)_ = 10.9; *p* = 5.0 × 10^–11^]. We also observed significant sex effects for Day 1 [*F*_(1_, _153)_ = 11.3; *p* = 9.7 × 10^–4^] and habituated locomotor behavior [*F*_(1_, _151)_ = 21.1; *p* = 9.0 × 10^–6^], acute response to cocaine [*F*_(1_, _151)_ = 4.6; *p* = 0.034], behavioral sensitization [*F*_(1_, _146)_ = 7.5; *p* = 0.007] and conditioned activation [*F*_(1_, _149)_ = 4.9; *p* = 0.028]. Test site differences were observed for Day 1 [*F*_(1_, _153)_ = 42.7; *p* = 8.8 × 10^–10^] and habituated locomotor behavior [*F*_(1_, _153)_ = 27.0; *p* = 6.5 × 10^–7^], initial sensitization [*F*_(1_, _150)_ = 13.8; *p* = 2.9 × 10^–4^] and behavioral sensitization [*F*_(1_, _146)_ = 16.5; *p* = 7.8 × 10^–5^]. Generally, mice tested at JAX were more active than mice tested at UNC. However, significant strain by testing site differences were detected for Day 1 [*F*_(7_, _153)_ = 4.4; *p* = 1.7 × 10^–4^] and habituated locomotor activity [*F*_(7_, _151)_ = 2.5; *p* = 0.017] and behavioral sensitization AUC [*F*_(7_, _146)_ = 3.0; *p* = 0.005[(Figures 5A–C). We also generated Pearson correlation coefficients of derived variables from CC and Founder mice tested at the two sites. The two behaviors with the lowest heritabilities, sensitization expression [Day 19–Day 11; *r*(6) = 0.34] and conditioned activation [Day 12–Day 2; *r*(6) = 0.62], were not significantly correlated between sites. The remainder of the derived variables had correlation coefficients ranging from 0.82 to 0.97 (Figure 4).

#### Collaborative cross replicability

**FIGURE 6:**
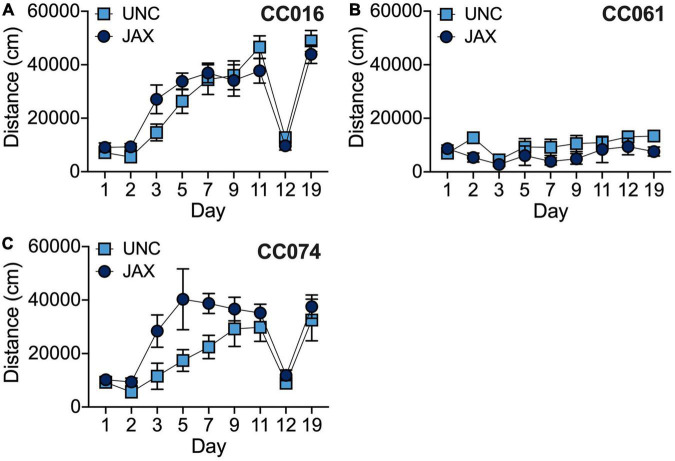
Comparison of behavioral sensitization in CC016/GeniUncJ (A), CC061/GeniUncJ (B) and CC074/UncJ (C) strains across test sites.

In addition to the founder strains, we also tested three CC strains at UNC. We chose two “high responding” strains (CC016/GeniUncJ, CC074/UncJ) and one “low responding” strain (CC061/GeniUncJ) (Supplementary Table 5). We used ANOVA to analyze locomotor activity with strain, test site, day and sex as independent factors. We observed significant strain effects [*F*_(2_, _254)_ = 86.3; *p* = 2.5 × 10^–29^] as well as significant strain by location [*F*_(2_, _254)_ = 6.5; *p* = 4.8 × 10^–4^], strain by day [*F*_(16_, _254)_ = 6.5; *p* = 3.1 × 10^–12^] and strain by sex [*F*_(2_, _254)_ = 9.5; *p* = 1.0 × 10^–4^] effects. CC061/GeniUncJ mice tested at JAX were significantly less active than both CC016/GeniUncJ and CC074/UncJ mice (both *p* < 0.001) and the two high-responding strains were not significantly different from each other. Similarly, CC061/GeniUncJ mice tested at UNC were significantly less active than both of the high responding CC strains (both *p* < 0.01), but CC074/UncJ mice tested at UNC were significantly less active than CC016/GeniUncJ mice (*p* < 0.01). Significant effects of day of testing were observed in both CC016 [*F*_(8_, _104)_ = 32.1; *p* = 8.1 × 10^–25^] and CC074 [*F*_(8_, _69)_ = 9.8; *p* = 5.7 × 10^–9^] strains but not CC061 [*F*_(8_, _81)_ = 1.2; *p* = 0.31] reflecting differences in response to cocaine observed among these strains (Figures 6A–C). A significant test site difference was observed for the CC074 strain [*F*_(1_, _69)_ = 13.2; *p* = 5.4 × 10^–4^] but not for CC016 [*F*_(1_, _104)_ = 0.78; *p* = 0.381] or CC061 [*F*_(1_, _81)_ = 3.9; *p* = 0.051] strains. This difference likely reflects increased response to cocaine on Days 3–7 in mice tested at JAX compared to those tested at UNC, but no significant test site by day interaction effects were observed for any strain.

## Discussion

The absence of effective pharmaceutical treatments for CUD has hindered our ability to effectively combat this devastating disorder. Gaps in our knowledge regarding the underlying etiology of CUD limit our ability to develop novel and efficacious pharmacotherapies. There is strong evidence for genetic factors that influence CUD risk (Goldman et al., 2005; Ducci and Goldman, 2012; Gelernter et al., 2014) but thus far, very few risk genes have been identified in human genome-wide association studies (Gelernter et al., 2014; Sun et al., 2020; Sherva et al., 2021). Preclinical studies in mice have been effective in identifying genetic loci and candidate genes that influence the activating and reinforcing effects of cocaine (Boyle and Gill, 2001, 2009; Gill and Boyle, 2003; Kumar et al., 2013; Dickson et al., 2016; Bagley et al., 2021), but the majority of these studies have been conducted on only a few inbred strain backgrounds, limiting the scope and generalizability of the findings. In fact, the most commonly used inbred strain, C57BL/6J, falls close to the middle of the phenotypic distribution for many of the sensitization variables that we studied (Supplementary Figures 1A–G). The use of the genetically diverse CC population allowed us to capture a broader range of phenotypes that can drive novel biological and mechanistic discoveries. It is clear that the phenotypic distribution of the CC strains exceeds that of the founder strains for almost all behaviors measured with the exception of locomotor activity on Days 1 and 2, measured prior to any drug exposures (Supplementary Figures 1A–G).

The ability to identify extreme responding strains is a significant benefit of measuring addiction-like behaviors and other phenotypes in the CC panel. We, and others, have identified extreme CC strains that can be used as models to study the genetic, biological and pharmacological mechanisms of disease (Rogala et al., 2014; Graham et al., 2015). Of particular note are the CC004/TauUncJ (CC004) and CC041/TauUncJ (CC041) strains (Figure 1) that were described in our previous publication (Schoenrock et al., 2020). CC041 mice are significantly less activated by an acute 20 mg/kg exposure to cocaine in comparison to CC004 mice. CC041 mice also fail to acquire intravenous cocaine self-administration and have a longer period and reduced amplitude in both molecular and behavioral circadian rhythms compared to CC004. Dopamine tissue content and dynamics are similar in the two strains (Schoenrock et al., 2020) suggesting that non-dopaminergic mechanisms may be driving behavioral differences. The expanded set of cocaine-induced locomotor sensitization data described here in the CC and founder population provides the opportunity to identify additional inbred CC strains with abnormal or extreme phenotypes to study mechanisms that contribute to cocaine-induced behavioral differences.

Behavioral sensitization occurs in several phases, including locomotor response to an initial exposure to the drug, augmented response upon additional exposures and finally, persistent elevation of the response even after a period of abstinence. Our locomotor sensitization protocol allows us to measure all of these phases and examine them as discrete variables. Examining correlations (or lack thereof) across different behavioral sensitization measures can inform us about genetic and phenotypic relationships across the phases of sensitization and inform future studies aimed at genetic mapping of discrete behaviors. Not surprisingly, the strongest correlations were observed between behavioral measures of locomotor activity following an injection of saline on Days 1 and 2, as well as on each of the consecutive days of cocaine treatment (i.e., Day 3 vs. Day 5, Day 7 vs. Day 9 etc.). Correlations between activity on any specific day and derived variables were generally lower (i.e., Day 1 vs. Day 3-Day 2, Day 11 vs. Day 5–Day 3) but still significant. Neither Day 1 nor Day 2 habituated locomotor response were significantly correlated with behavioral sensitization measures (Day 5–3, AUC) suggesting that locomotor activity itself does not solely predict the locomotor-sensitizing properties of cocaine in this population. However, it is likely that strain differences in overall response to cocaine across all of the test days can be attributed, at least in part, to strain-specific locomotor activity. This finding isn’t surprising as the relationship between locomotor activity and psychostimulant-induced locomotor activation has been repeatedly observed in our laboratory (Wiltshire et al., 2015; Roberts et al., 2018) and reported by others (Deroche et al., 1993; Mathews et al., 2010; Thomsen and Caine, 2011). Sensitization expression (Day 19–Day 11) does not correlate with any other behavioral measure and also has fairly low heritability (H^2^ = 0.16). Locomotor response to cocaine on Day 19 was remarkably consistent in comparison to Day 11 across all strains even after 7 days of abstinence and regardless of the level of locomotor sensitization across cocaine exposure days (Supplementary Table 5A). Our data suggest that in this population, genetic influences are more important in the initiation or development phases and not in the maintenance or expression of the sensitized response. These results inform our interpretation of the role genetics may play in cocaine-induced neural adaptations. Our data could inform strategies using genetic information to develop therapeutic interventions targeted toward the development of addiction and ongoing drug use vs. relapse.

**FIGURE 7:**
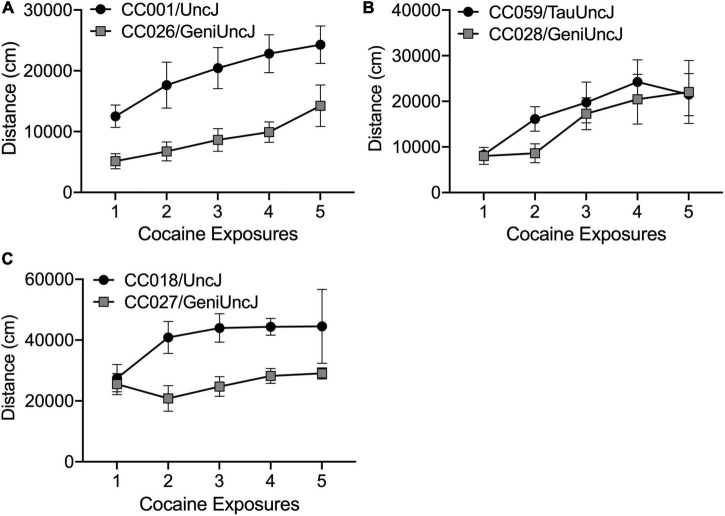
Different patterns of sensitized cocaine locomotor behavior in CC strains of interest. Cocaine exposures 1–5 correspond to Days 3–11 in the sensitization protocol. (A) CC001/UncJ and CC026/GeniUncJ show similar rates of sensitization but differ in overall locomotor response; (B) CC059/TauUncJ mice exhibit a robust sensitization response across the first four exposures while CC028/GeniUncJ mice don’t begin to show a sensitized response until the third exposure and (C) the maximal locomotor response in CC018/UncJ mice is reached by the second dose whereas CC027/GeniUncJ mice show the highest response after a single exposure and locomotor activity does not increase upon additional exposures.

The derived sensitization variables (described in Table 2) are discrete behavioral measures necessary for cross-strain comparisons and statistical analyses as well as future mapping studies in the outbred DO population. However, these variables do not fully capture the diversity of behavioral responses across the CC and founder strains. Closer examination of behavior across the 19-day sensitization assay can highlight distinct sensitization patterns [as previously described by Smith et al. (2016)], that suggest acute differences in drug response (Figure 7A), rate of sensitization (Figure 7B) and differences in maximal response to cocaine (Figure 7C). However, we note that differences in drug response may be dose-specific and our study was limited to a single dose. Strain differences reported here must be more rigorously tested with larger group sizes and additional doses of cocaine. Assessing dose-response in specific strains of interest is necessary prior to moving forward with mechanistic studies aimed at explaining behavioral differences.

Phenotype replicability is an important consideration in preclinical studies and has been discussed in great detail in the field of behavioral genetics (Crabbe et al., 1999; Kafkafi et al., 2018). The inability to replicate behaviors across experimental sites, under different protocols or across time can limit the generalizability and translatability of findings and has broader implications for the overall stability and robustness of commonly used behaviors. We did observe differences in behavior in founder strains across sites, but these were mostly main effects that were manifest as parallel shifts in behavior across all strains. The traits that were the least reliably measured within a lab were also the least replicable across labs. Strain by test site interactions were observed for a few phenotypes (Figure 5) but the correlation between behaviors was remarkably strong between sites (Figure 4) even though there were site-specific differences in testing protocol, including prior test history.

We also observed replication among the CC strains for which we have data from both sites (Figures 6A–C). Strain by test site interactions mainly appear to be driven by behavioral variability for locomotor activity on Day 2 in CC061 mice (Figure 6B). We also observe differences in the rate of the sensitization response in CC074 mice (Figure 6C), although sensitized locomotor activity on the final day of the sensitization protocol (Day 11) is remarkably well replicated across sites. Variability in behavior observed across sites could reflect site-specific differences in the protocol, animal handling or strain-specific sensitivities to environmental influences on cocaine-induced locomotor activity and behavioral sensitization. Further studies in these diverse, yet genetically well-defined inbred mouse strains may provide important insights into gene by environment interactions that drive addiction-relevant behaviors. Regardless, the results of our replication studies are encouraging considering the differences in testing protocols at UNC vs. JAX (group vs. single housing, test naïve mice vs. previous exposure to testing, etc.) and the procurement of mice from different facilities which introduces additional environmental factors including exposure to different diets during development and post-weaning, transportation etc. Overall, we found that the pattern of cocaine-induced behaviors for a given strain (low vs. high responder) were largely replicated across test sites (Figures 5, 6). We note that our replication experiments were limited to only 3 CC strains and the number of mice tested at JAX for some strains is quite low. Replication studies on a larger number of CC strains with increased sample sizes are warranted to make more definitive conclusions about overall and strain-specific reproducibility of behavior across sites.

Our strain survey of cocaine-induced behavioral sensitization in the CC population provides a unique data resource that will be of great use to the addiction genetics community. Individual strains can be readily accessed for use as disease models for studying neurobiological and pharmacological mechanisms of addiction. Additionally, our sensitization phenotypes can be compared with other addiction-relevant phenotypes collected in the CC. We have already observed that high and low responding strains, CC004 and CC041, differ for acquisition of cocaine self-administration (Schoenrock et al., 2020). Measures of impulsivity have been linked with SUD and addiction behaviors in humans and animal models [as reviewed by Jentsch et al. (2014)]. Bailey et al. (2021) observed high impulsivity behaviors in CC018 and CC026. We have observed that, compared to some other strains, CC018 and CC026 appear to have a higher maximal response to cocaine and a lower rate of sensitization, respectively (Figure 6). Our data also offer a unique opportunity to study the relationship between psychomotor and incentive sensitization in a genetically and phenotypically diverse population. The incentive sensitization theory of addiction posits that drug-induced sensitization of the mesocorticolimbic circuitry in the brain alters incentive-motivational processes, thereby imbuing drugs and drug-related stimuli with enhanced salience and ability to elicit behavior. For the most part, the basic tenets of this theory have been upheld in the literature (Robinson and Berridge, 2008). Although behavioral sensitization is often used as an indirect measure of changes in the mesocorticolimbic circuitry in the brain, direct comparisons of psychomotor and incentive sensitization are largely absent in the literature. Measuring incentive sensitization in genetically stable, inbred mouse strains with known phenotypic variation in psychomotor sensitization would allow us to understand the relationship between locomotor changes in response to repeated cocaine exposure and changes in the incentive salience of the drug. Understanding the relationship between addiction-relevant behaviors will be useful for designing and interpreting data from mechanistic studies.

The heritability observed for most of the sensitization behaviors also indicates that mapping strategies to identify genetic loci and, ultimately, addiction risk genes, will be fruitful. Genetic mapping in the DO population is currently underway in the CSNA. Identification of risk genes in using this rodent-based assay will inform our knowledge of the mechanisms and pathways that affect differential responses to repeated cocaine exposures and generate novel hypotheses regarding the path from initial drug use to a CUD.

## Data availability statement

The datasets presented in this study can be found in online repositories. The names of the repository/repositories and accession number(s) can be found below: https://phenome.jax.org/projects/CSNA03.

## Ethics statement

This animal study was reviewed and approved by the Institutional Animal Care and Use Committees at the University of North Carolina at Chapel Hill and the Jackson Laboratory.

## Author contributions

EC, JJ, LT, SAS, SJS, LG, LR, and VP contributed to the conception and design of the study. SAS, AO, and ML collected all the data. SAS, VP, and HH performed the statistical analysis. SAS and LT wrote the first draft of the manuscript. All authors contributed to the manuscript revision, read, and approved the submitted version.

## Footnotes

1
phenome.jax.org
